# Short-term outcomes of aortic valve neocuspidization for various aortic valve diseases

**DOI:** 10.1016/j.xjon.2021.08.027

**Published:** 2021-08-26

**Authors:** Gregory Khatchatourov, Mathieu van Steenberghe, Doris Goy, Mathieu Potin, Javier Orrit, François Perret, Nicolas Murith, Jean-Jacques Goy

**Affiliations:** a Department of Cardio-Surgery, Clinique Cecil, Lausanne, Switzerland; b University Hospital of Geneva, Geneva, Switzerland

**Keywords:** cardiac surgery, Ozaki procedure, aortic valve neocuspidization, aortic valve disease, autologous glutaraldehyde fixed pericardium, aortic valve reconstruction, AR, aortic regurgitation, AS, aortic stenosis, AV, aortic valve, AVA, aortic valve area, AVD, aortic valve disease, AVneo, aortic valve neocuspidization, AVR, aortic valve replacement, BAV, bicuspid aortic valves, CPB, cardiopulmonary bypass, IE, infective endocarditis, MAVRE, major adverse valve related event, NYHA, New York Heart Association, PPG, peak pressure gradient, TEE, transesophageal echocardiography

## Abstract

**Objectives:**

Bioprosthetic valve deterioration remains a major limitation following aortic valve replacement. Favorable results have been reported with an autologous pericardium aortic valve neocuspidization.

**Methods:**

Seventy patients (31 women and 39 men) (mean age, 62 ± 12 years) with aortic stenosis (n = 52 [74%]) or aortic regurgitation (n = 18 [26%]) underwent the aortic valve neocuspidization procedure. Thirty-four patients (49%) had a tricuspid valve, 35 (50%) had a bicuspid valve, and 1 (1%) had a monocuspid valve. European System for Cardiac Operative Risk Evaluation and Society of Thoracic Surgeons scores were, respectively, 2.2% ± 2% and 2.0% ± 1.8%. Four patients (6%) had active endocarditis and 2 (3%) had endocarditis sequelae. One patient (1%) had fibroelastoma. A combined procedure was performed in 33 patients (46%).

**Results:**

The follow-up period was 24 ± 12 months. One patient (1%) died in hospital and 1 patient (1%) underwent conventional valve replacement for significant aortic regurgitation. Postoperative peak and mean pressure gradients were respectively 14 ± 5 and 8 ± 3 mm Hg. Aortic valve area was 2.5 ± 0.6 cm^2^. During follow-up, no patients died. Reintervention occurred in 2 patients (3%). At last follow-up, peak pressure gradient was 13 ± 7 mm Hg, mean pressure gradient was 7 ± 4 mm Hg, and aortic valve area was 2.3 ± 0.7 cm^2^. There was 1 recurrence of moderate aortic stenosis (1%). All patients were in New York Heart Association functional class I (90%) or II (10%). Freedom from major valve-related events was 92.1%, (98.5% for death, 95.2% for reintervention, and 95.2% for endocarditis).

**Conclusions:**

In our experience, the midterm outcomes of the aortic valve neocuspidization procedure with autologous glutaraldehyde fixed pericardium were acceptable for survival, operative risk and valve-related complications, for our all-comer patient population with various aortic valve diseases.

## Graphical abstract

**Figure:**
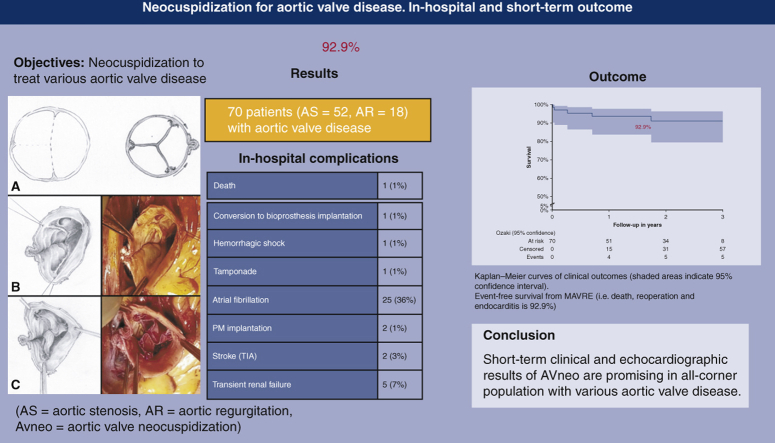

**Figure:**
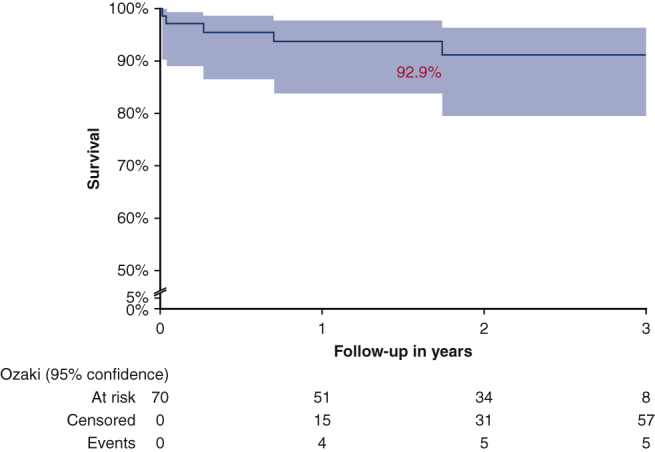
Event-free survival curve after AVneo.

**Central Message**

Short-term clinical and echocardiographic results of AVneo are promising in an all-comer population with various aortic valve diseases.

**Perspective**

We report the results of AVneo using glutaraldehyde-treated autologous pericardium in an all-comer population. The midterm results with a mean follow-up of 24 months (longest follow-up, 50 months) were acceptable without serious complications. The echocardiographic follow-up showed persistent hemodynamic parameters benefit over time.

See Commentaries on pages 203 and 205.

There is no ideal aortic valve (AV) substitute. AV replacement (AVR) by biological or mechanical prosthesis remains the gold standard treatment for adults with aortic stenosis (AS) or aortic replacement (AR), whereas valve repair or sparing procedures are preferred for AR and root aneurysms for younger patients in experienced centers.^1^

Nonetheless, prosthesis implantation has limitations related to structural valve deterioration and need for long-term anticoagulation. Bioprostheses made with heterologous tissue are prone to degeneration after 10 to 15 years in adults and more rapidly in young patients.^2^ The major mechanism of degeneration is the immunologic reaction against xenoantigens in the graft. However, young patients have both higher phosphocalcic metabolism and more reactive immune systems that lead to earlier bioprosthesis degeneration. Mechanical valves are often recommended to this population for better durability, but this procedure exposes young patients to long-term anticoagulation therapy and its related complications.^3^^,^^4^

AV repair and valve-sparing procedures are attractive approaches because they overcome the previously mentioned limitations. In the early era of cardiac surgery, AV repair was performed with native valve leaflets by means of various techniques.^5^, ^6^, ^7^, ^8^ Different types of tissue, including fascia lata, were used with mitigated results. Among these, autologous pericardium showed the best results in terms of durability for reconstruction in adult and pediatric populations.^9^ However, these repair and sparing procedures are limited by AV leaflet quality, especially when there are degenerative structural modifications and calcifications.

Duran and colleagues^10^, ^11^, ^12^, ^13^ described a technique of complete AVR with autologous tissue. The overall rate of freedom from reoperation was 47% at 16 years.^13^ Recently, Ozaki and colleagues^14^, ^15^, ^16^ and Takahashi and colleagues^17^ reported encouraging results with a new procedure for AVR combining use of autologous tissue and complete native valve removal. Reconstruction is by means of a separate 3-leaflet design made of autologous pericardium. Sizing is based on the intercommisural distance measurement that gives the neoleaflets free margin lengths, thus providing perfect neoleaflets coaptation at the commissural level (higher than in native AV). This simple geometrical principle allows hemodynamically reproducible results and can be used to treat a wide spectrum of AV diseases (AVDs), including AS, AR, infective endocarditis (IE), prosthetic valve endocarditis, and fibroelastoma. Whereas root aneurysm is initially limiting, a recent report has shown feasibility and efficacy in root replacement combined with AV neocuspidization (AVneo).^18^ Moreover, this technique offers the advantage of using only autologous tissue and also allows attainment of systolic/diastolic root dynamics and effective orifice areas nearly similar to physiologic conditions.^19^^,^^20^

Therefore, in 2016, after following live cases and dry lab training, we started an AVneo program in our institution. This study reports the clinical and hemodynamic outcomes at short-term of our AVneo procedure for a wide spectrum of AVDs in an all-comer population.

## Methods

### Population

All consecutive patients referred to our clinic for AV surgery were considered candidates for the AVneo procedure. Exclusion criteria were redux operation and aortic root aneurysm. A decreased ejection fraction was not an exclusion criteria. The patients were fully informed of this new technique and of the conventional AVR approaches. They were operated according to their personal preferences.

The study was conducted in compliance with the ethical requirements of Swiss law on the quality control of surgical procedures (RO 1995 1328, Loi fédérale du 18 mars 1994 sur l'assurance-maladie [LAMal] article 58). Patients were informed of the procedure and required to sign an informed consent for surgery. Individual written consent to publish their study data was also obtained. All patients had significant clinical AVD, in the form of either AS or AR. Patients with concomitant AS and AR were assigned to the AS group because the stenotic effect was predominant. The preoperative investigations were not different from those usually performed before an AVR. All patients underwent TTE, coronarography, and right and left cardiac catheterism. Angio computed tomography or magnetic resonance imaging were performed in patients with aneurysm or bicuspid AV (BAV).

### Procedure

After full sternotomy, the anterior pericardium was freed from surrounding fat and fibrous tissues and then harvested. Additional preparation on the back table included immersion in 0.6% buffered glutaraldehyde solution for 10 minutes followed by a 6-minute rinse in 0.9% sodium chloride solution repeated 3 times. Lateral pericardium can also be used but its harvest is limited by the presence of the phrenic nerve. In case of redo for IE after AVneo replacement, the diseased leaflet was replaced by decellularized bovine pericardium (CardioCel; Admedus, Brisbane, Australia).

Normothermic cardiopulmonary bypass (CPB) was instituted after standard cannulation. Cardioplegic arrest was achieved via antegrade followed every 15 to 20 minutes by intermittent retrograde cold blood perfusion (St Thomas's and blood, 1:4 at 4°); hot-shot induction is given immediately before unclamping, according to our local protocol. Partial aortotomy is the usual approach at the sinotubular junction level 1.5 cm above the right coronary ostium. In small aortic roots or in bicuspid valves, to improve exposure, complete aortotomy was performed at the same level. The AV was excised and any remaining calcifications of the root, were removed using a Cavitron Ultrasonic Surgical Aspirator (SonoSurg; Olympus, Tokyo, Japan).

**Figure 1:**
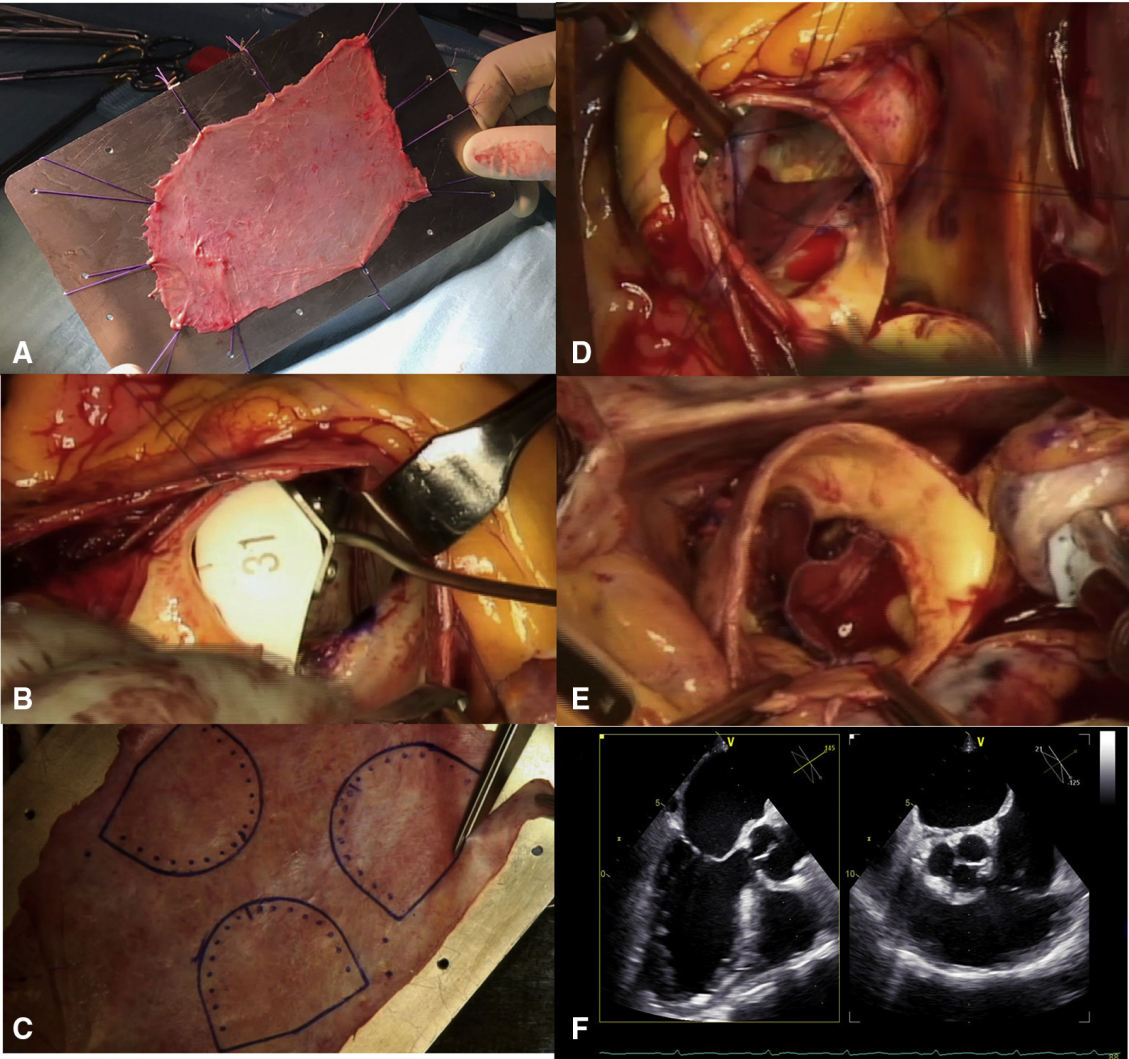
Intraoperative views of the aortic valve neocuspidization procedure. A, Pericardium preparation. B, Neoleaflet sizing with the dedicated sizer using intercommissural distance as reference. C, Cutting the autologous pericardium according to templates. D, Implantation of the neoleaflet. E, Neoaortic valve after procedure completion. F, Final echocardiographic view.

**Figure 2:**
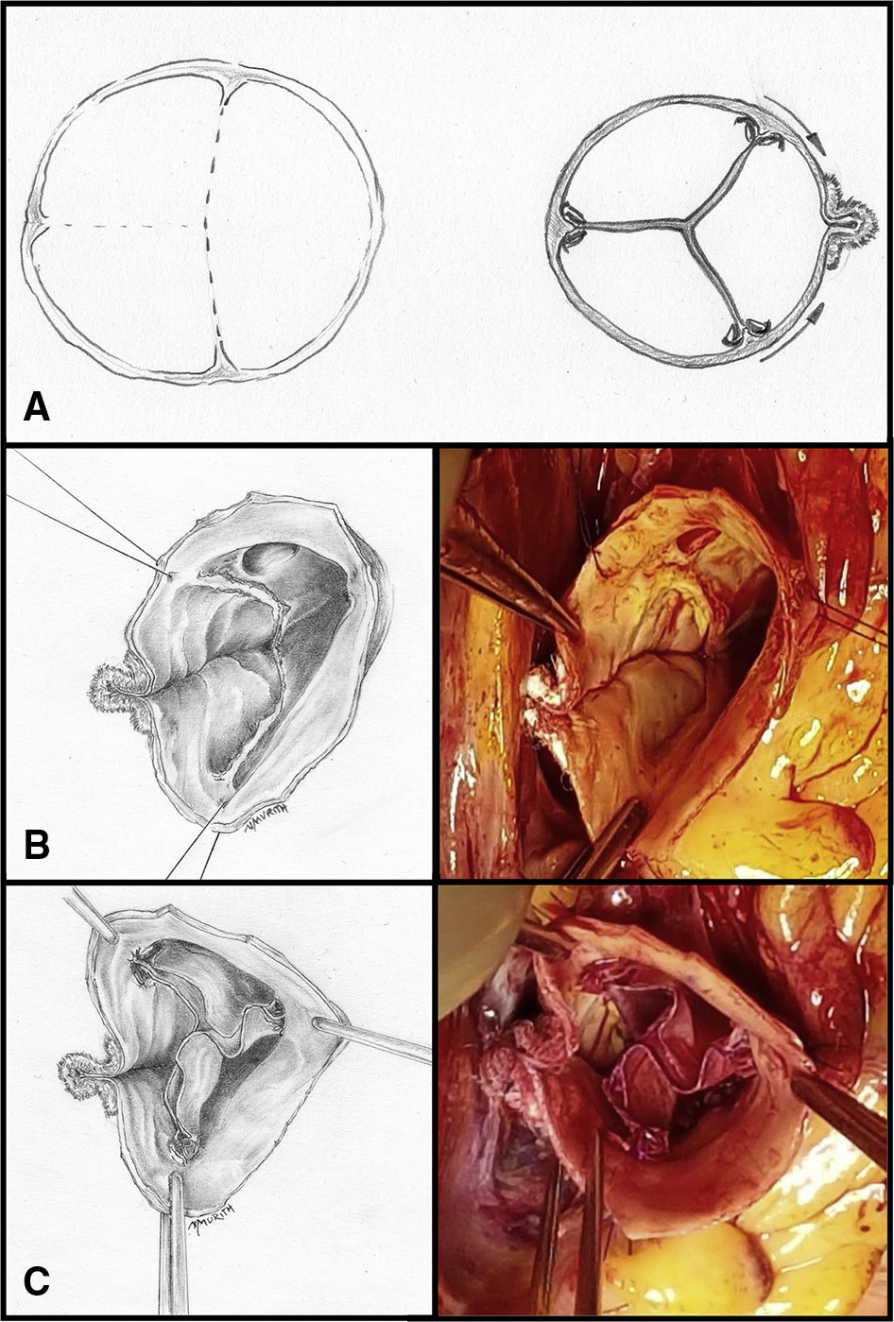
Our sinus plication in Sievers type-I left/right BAV. A, The drawings illustrate a Sievers type-I left/right BAV and a symmetrical aortic valve neocuspidization procedure (AVNeo) with longitudinal noncoronary sinus plication for the same. B, Drawing and intraoperative view of noncoronary sinus plication from the nadir to the commissural level. C, Drawing and intraoperative view of AVNeo with noncoronary sinus plication shows the symmetrical coaptation of the 3 neoleaflets at the same level. *BAV*, Bicuspid aortic valve.

Sizing is among the most important aspects of this procedure (Figure 1). The intercommissural distance was used as reference for adequate sizing and measured with dedicated sizers (JOMDD, Tokyo, Japan). As recommended by Ozaki and colleagues^14^, ^15^, ^16^ we accepted a 1-size difference between the 3 neoleaflets; otherwise, a new commissure was created to reach a more symmetrical anatomy. The autologous pericardial leaflets were cut out using special templates (JOMDD) according to the sizing and then sutured to the annulus starting from each nadir to the commissure level, using a 4–0 monofilament suture (Figure 1). Commissures were secured by additional 4–0 stitches. Free margins of the neoleaflets had to ensure symmetrical coaptation at the same commissural level (Figure 1). In BAVs, tricuspidization and creation of a neocommissure had to be performed. In Sievers type I left/right BAV, we reached symmetry by longitudinal plication of the noncoronary sinus (Figure 2). The sinus was dissected from outside reaching the nadir of the annulus. Two strips of polytetrafluoroethylene felt 0.5 to 1.0 cm wide were applied from the outside starting the plication from the annulus to the level of the sinotubular junction with 4–0 monofilament suture. After weaning off CPB, transesophageal echocardiography (TEE) was performed to confirm the result of the procedure. Video 1 summarizes the various steps of the procedure. Patients were discharged taking aspirin 100 mg/day. Anticoagulation therapy was given only when clinically needed.

### Data Collection

Surgery was performed using continuous TEE monitoring. Follow-up was conducted as required by good clinical practice rules following cardiac surgery. A control transthoracic echocardiography scan was performed at discharge, at 3 months, and annually by the referring cardiologist. Data were collected by the clinic study nurse and maintained on a computerized data bank. Standard demographic, clinical, TEE, and transthoracic echocardiography data for cardiovascular studies were recorded. Symptoms were classified according to New York Heart Association (NYHA) functional class.

### End Points

The primary end point was incidence of major adverse valve-related events (MAVRE) defined as cardiac death, reoperation, and IE. Secondary end points were occurrence of significant AR (grade III or IV), peak pressure gradient (PPG) >30 mm Hg, AV area (AVA) <1.0 cm^2^, and NYHA functional class III or IV status.

### Follow-up

Patients were discharged taking aspirin 100 mg/day. Anticoagulant therapy was given only when clinically needed (eg, patients with atrial fibrillation). Other medication was administered as required. Clinical examination with TEE was performed before discharge and annually thereafter with measurement of ejection fraction, AVA, AVR grade, pressure gradients, and pulmonary pressures. Symptoms were classified according to NYHA functional class.

### Statistical Analysis

Data are presented as mean ± standard deviation for continuous variables and number (%) for categorical variables. The 2 groups (preoperative and during-follow-up echocardiographic data) were evaluated by a paired Student *t* test. Kaplan-Meier analysis assessed the cumulative proportion of patients without MAVRE during the follow-up. All analyses were performed using the Stata statistical package (StataCorp LLC, College Station, Tex).

## Results

### Preprocedural Data

Baseline demographic and echocardiographic data are presented in Table 1. During the study period, 436 patients underwent AVR in our institution. Of them, 70 patients (31 women and 39 men) with a mean age of 62 ± 11 years underwent AVneo intervention. The mean surgical annulus diameter was 24 ± 4 mm and the mean ejection fraction was 62% ± 10%. Forty-five patients (64%) had AS, 18 patients (26%) had AR, and 7 patients (10%) had combined AS and AR (Table 1). Valve morphology was bicuspid in 35 patients (50%) and tricuspid in 34 patients (49%) (Table 1). One patient (1%) had a monocuspid valve. The average European System for Cardiac Operative Risk Evaluation score was 2.2 ± 2 and average Society of Thoracic Surgeons score was 2 ± 1.8.

**Table 1:** Demographic characteristics and baseline echocardiographic data

Characteristic	Total (N = 70)
Mean age (y)	62 ± 12
Male	39 (56)
Female	31 (44)
Weight (kg)	76 ± 13
Height (cm)	169 ± 10
BMI	26 ± 4
AS	45 (64)
AR	18 (26)
AS + AR	7 (10)
Smoker	14 (20)
Former smoker	14 (20)
Dyslipidemia	37 (54)
Hypertension	41 (58)
Family history of CAD	14 (20)
Diabetes	12 (17)
COPD	12 (17)
Previous stroke	4 (6)
TIA	2 (3)
Renal failure	1 (1)
CAD	13 (19)
Previous PCI	3 (4)
Previous MI	4 (4)
Peripheral vasculopathy	6 (9)
Previous CVS	2 (4)
BP systolic (mm Hg)	136 ± 15
Diastolic (mm Hg)	75 ± 12
Heart rate (bpm)	80 ± 9
Sinus rhythm	66 (94)
AF or flutter	4 (6)
EuroSCORE II (%)	2.2 ± 2
STS score (%)	2 ± 1.8
Mean EF (%)	62 ± 10
Unicuspid valve	1 (1)
Bicuspid valve	35 (50)
Tricuspid valve	34 (49)
Annulus diameter (mm)	24 ± 4
PPG (mm Hg)	66 ± 20
MPG (mm Hg)	42 ± 13
AVA (cm^2^)	0.72 ± 0.2
PAPs (mm Hg)	33 ± 11
AR grade	
I	0
II	8
III	11
IV	6

Values are presented as mean ± standard deviation, n (%), or n. *BMI*, Body mass index; *AS*, aortic stenosis; *AR*, aortic regurgitation; *CAD*, coronary artery disease; *COPD*, chronic obstructive pulmonary disease; *TIA*, transient ischemic attack; *PCI*, percutaneous coronary intervention; *MI*, myocardial infarction; *CVS*, cardiovascular surgery; *BP*, blood pressure; *AF*, atrial fibrillation; *EuroSCORE II*, European System for Cardiac Operative Risk Evaluation II; *STS*, Society of Thoracic Surgeon; *EF*, ejection fraction; *PPG*, peak pressure gradient; *MPG*, mean pressure gradient; *AVA*, aortic valve area; *PAP*, pulmonary arterial gradient.

Four patients (6%) had active IE with hemodynamic influence. Echocardiographic measurements showed 3 patients with AR grade II, 9 patients with AR grade III, and 6 patients with AR grade IV. Mean pulmonary arterial pressure was 33 ± 11 mm Hg. PPG was 66 ± 20 mm Hg, mean pressure gradient was 42 ± 13 mm Hg, mean AVA was 0.72 ± 0.2 cm^2^, and mean pulmonary arterial pressure was 33 ± 11 mm Hg. AVneo alone was performed in 38 patients (54%) and association with 1, 2, or 3 additional procedures was reported in 32 patients (46%) (Table 2).

**Table 2:** Description and incidence of surgical procedures

Procedure	Total (N = 70)
AVneo procedure alone	38 (53)
AVneo + AAR	10 (15)
AVneo + AAR + CABG	1 (1)
AVneo + AAR + myomectomy	3 (4)
AVneo + AAR + auricule ligation + tricuspid annuloplasty	1 (1)
AVneo + ventricular myomectomy	6 (9)
AVneo + maze procedure	1 (1)
AVneo + CABG	5 (9)
AVneo + mitral valve repair	3 (4)
AVneo + mitral repair + CABG	1 (1)
AVneo + mitral valve replacement	1 (1)

Values are presented as n (%). *AVneo*, Aortic valve neocuspidization; *AAR*, ascending aortic replacement; *CABG*, coronary artery bypass grafting.

### Periprocedural Data

The mean aortic cross clamp time was 143 ± 33 minutes and the mean CBT was 157 ± 37 minutes. Mean CBT and crossclamp time were shorter in patients without combined procedure and no tricuspidization. As expected, they were longer when tricuspidization was associated with a combined intervention as shown in Table 3. All patients underwent the intended preprogrammed AVneo intervention and all procedures were completed as scheduled.

**Table 3:** Mean cardiopulmonary bypass time (*CBT*) and crossclamp time (*CCT*) for the various procedures∗

Procedure	No tricuspidization, no combined procedure	No tricuspidization, combined procedure	Tricuspidization, no combined procedure	Tricuspidization, combined procedure
CBT (min)	136 ± 23	166 ± 53	154 ± 22	179 ± 38
CCT (min)	124 ± 20	150 ± 44	142 ± 21	162 ± 35

Values are presented as mean ± standard deviation.

∗ The first column shows the mean duration for standard isolated procedures of aortic valve replacement in our center. The second column shows the mean duration for combined procedures with aortic valve replacement in our center. The third column shows isolated aortic valve neocuspidization procedures and the fourth column shows combined procedures with aortic valve neocuspidization in our series.

### Postprocedural Data

#### Clinical outcomes

In-hospital stay duration was 14 ± 6 days (Table 4). Minor complications occurred in 43 patients (61%). Two patients (3%) underwent pacemaker implantation, 1 patient (1%) had a tamponade requiring drainage, and 1 patient (1%) had severe gastrointestinal bleeding due to a gastric ulcer. Two patients (3%) had transient ischemic attacks with complete recovery, and 5 patients (7%) experienced transient renal failure with eventual full recovery of renal function. The most frequent benign complication was atrial fibrillation in 25 patients (36%). Complete left bundle branch block occurred in 9 patients, 7 in the AS group, and 2 in the AR group.

**Table 4:** In-hospital and long-term outcomes and complications

Outcome	Total (N = 70)
In-hospital stay (d)	14 ± 6
Death	1 (1)
Conversion to bioprosthesis implantation	1 (1)
Hemorrhagic shock	1 (1)
Tamponade	1 (1)
Atrial fibrillation	25 (36)
PM implantation	2 (1)
Stroke/TIA	2 (3)
Transient renal failure	5 (7)
Myocardial infarction	0
New LBBB	9 (10)

Values are presented as mean ± standard deviation or n (%). *PM*, Pacemaker; *TIA*, transient ischemic attack; *LBBB*, left bundle branch block.

Major complications occurred in 2 patients (3%). One patient (1.5%) with acquired immunodeficiency syndrome and effective immunosuppression died from ongoing sepsis, and 1 patient (1.5%) was reoperated on for significant AR due to commissural distortion at day 10 and received a bioprosthetic implant. In 3 patients a transient treatment with colchicine was introduced due to the intensity of the inflammatory response. There was no myocardial infarction, severe arrhythmia or resuscitation during the in-hospital stay. Thirty-day mortality was 1.4%.

#### After discharge

The median follow-up period was 24 ± 12 months (cumulative follow-up of 140 patient-years) (Table 5). No patient was lost during the follow-up period and follow-up is complete. There was no death. Reoperation had to be performed on 2 patients (3%). IE resulted in reoperation for 1 patient (1%) after 8 months. He underwent a new AVneo procedure for 1 leaflet replacement with decellularized bovine pericardium (CardioCel, Admedus, Brisbane, Australia) with an uneventful outcome. The second patient (1%) showed moderate-to-severe AR with suture dehiscence on one neoleaflet at 3 months after surgery. The valve was replaced by a mechanical aortic prosthesis. Reoperations after AVneo were not more complex than usual. The aortic annulus had less fibrotic modifications than after prosthetic valve replacement. There were no stroke or pacemaker implantations during follow-up. Two patients (3%) had atrial fibrillation episodes.

**Table 5:** Population clinical outcomes at follow-up for primary and secondary end points (n = 69)

Outcome	Total (n = 69)
Death	0
Endocarditis	2 (3)
Reoperation	2 (3)
Stroke	0
AF	2 (3)
Pacemaker implantation	0
NYHA functional class I	62 (90)
NYHA functional class II	7 (10)
NYHA functional class III	0
NYHA functional class IV	0

Values are presented as n (%). *AF*, Atrial fibrillation; *NYHA*, New York Heart Association.

#### End points

**Figure 3:**
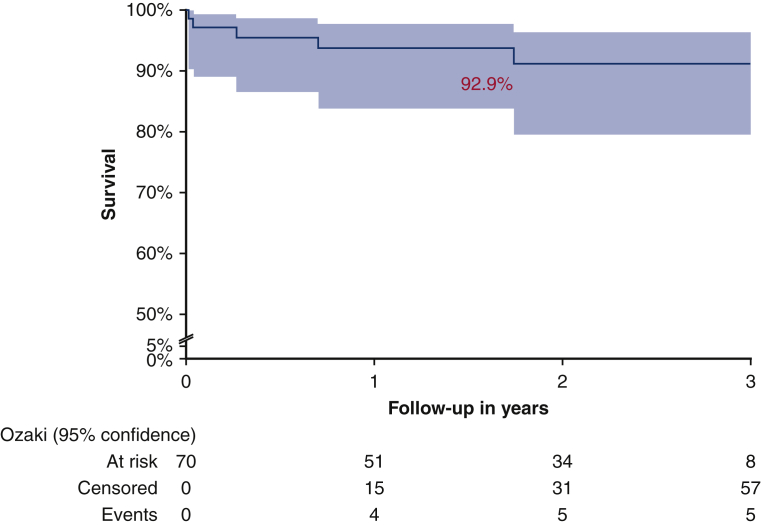
Kaplan-Meier curves of clinical outcomes (*shaded areas* indicate 95% confidence interval). Event-free survival from major adverse valve related event (ie, death, reoperation, and endocarditis was 92.9%).

Primary end point analysis showed actuarial freedom from MAVRE event-free survival of 92.9% (98.5% for death, 95.2% for reoperation, and 95.2% for IE) (Figure 3).

Secondary end point analysis showed 62 patients (88%) in NYHA functional class I, and 7 patients (12%) in NYHA functional class II. No patients were in NYHA functional class III or IV. Only 1 patient had a positive secondary end point with AVA <1.0 cm^2^.

#### Echocardiographic outcomes

Table 6 summarizes echocardiographic data before AVneo procedure, at hospital discharge, and after follow-up. Patients were followed on a yearly basis as recommended by the European Society of Cardiology after valve surgery following a first control 6 months after the intervention.^21^

**Table 6:** Echocardiographic data before surgery and at hospital discharge and follow-up

Data point	Preoperative	Discharge (n = 69)	Follow-up (n = 69)	*P* value between preoperation and follow-up
Mean EF (%)	62 ± 10	63 ± 10	62 ± 9	
PPG (mm Hg)	66 ± 20	14 ± 5	13 ± 7	<.0001
MPG (mm Hg)	42 ± 13	8 ± 3	7 ± 4	<.0001
AVA (cm^2^)	0.72 ± 0.2	2.5 ± 0.6	2.3 ± 0.7	<.0001
PAPs (mm Hg)	33 ± 11	29 ± 6	27 ± 5	.003
AVA <1 cm^2^		0	1	
PPG >30 mm Hg	31	0	6	<.001
AVR none		54 (78)	54 (78)	
AVR grade I		15 (22)	12 (17)	
AVR grade II		0	3 (5)	
AVR grade III or IV		0	0	

Values are presented as mean ± standard deviation or n (%). *EF*, Ejection fraction; *PPG*, peak pressure gradient; *MPG*, mean pressure gradient; *AVA*, aortic valve area; *PAP*, pulmonary arterial gradient; *AVR*, aortic valve replacement.

Mean PPG decreased significantly at discharge and at last follow-up in comparison to the preoperative assessment with 14 ± 5 mm Hg at discharge and 13 ± 7 mm Hg at last follow-up compared with 66 ± 20 mm Hg and 42 ± 13 mm Hg preoperatively (*P* < .01). Mean AVA increased significantly after the procedure with 2.5 ± 0.6 cm^2^ at discharge and 2.3 ± 0.7 cm^2^ at last follow-up compared with 0.72 ± 0.2 cm^2^ preoperatively (*P* < .01). Mean pulmonary arterial pressure decreased from 33 ± 11 mm Hg at preoperative assessment to 29 ± 6 mm Hg at discharge and 27 ± 5 mm Hg at last follow-up (*P* < .05). At discharge, 54 patients had no AR (78%) and 15 patients (22%) had AR grade I. No AR grade II, III, or IV was reported in this group at discharge. At follow up 3 patients (5%) had progressed from AR grade I to grade II. The 54 patients without AR at discharge remain free AR at last follow-up.

## Discussion

The aim of this study was to analyze and report our results of the promising AVneo procedure in a wide range of AVDs in both old and young patients. Our results are favorable in terms of mortality, transaortic valve gradients, freedom from MAVRE, and recurrence of AS. This correlates well with previous reports and confirms reported advantages of the AVneo procedure over more conventional prosthesis-based remedies, including avoidance of prosthetic ring and foreign material that significantly reduces the AVA and compromises dynamics of the aortic root,^20^ and use of autologous material that precludes immune response and early degeneration and could provide better resistance to infections. However, this last point needs to be proven.

Preliminary data published by Ozaki and colleagues^16^ on a cohort of 88 patients treated between 2007 and 2009 showed good clinical results with 100% of patients free from reoperation, excellent echocardiographic outcomes with no AS or AR recurrence, and a better quality of life without anticoagulation therapy.

Further reports confirm these preliminary data with follow-up durations up to 5 years.^14^ Only 1% of patients required reoperation for severe AR or IE. At follow-up of 73 months, 97% of patients were free from reoperation.

Indeed, the largest published series by Ozaki and colleagues^15^ confirms these benefits in 850 patients with a mean follow-up of almost 4 years. Actuarial freedom from death, cumulative incidence of reoperation, and recurrent moderate or severe AR were, respectively, 86%, 4%, and 7%.

In our early experience, reoperation was needed in 1 patient because of moderate AR induced by commissural distortion. So, even if the AVneo is reproducible, strict technical recommendations have to be followed, especially during the learning stage, which is longer for bicuspid valve than for tricuspid valve morphology. For surgeons who are familiar with aortic surgery, approximately 20 cases of AVneo seems to be a reasonable number to perform the procedure safely.

A second case of reoperation at 5 months was due to suture tear with consecutive AR in a case of AVneo tricuspidization for BAV. The tear was located between the neoleaflet and its insertion in noncoronary sinus. Therefore, as previously mentioned, for Sievert type 1 left/right BAV we performed longitudinal application of noncoronary sinus to keep the suture line at the native fibrous leaflet insertion level, thus avoiding tear risk of the more fragile sinus tissue (Figure 2).

Data from other centers is somewhat limited; however, encouraging results have been reported recently in smaller series of 30, 52, 71, and 103 patients.^31^, ^22^, ^23^, ^24^ These studies are consistent and show good midterm results with low incidence of reoperation and low echocardiographic pressure gradients. Our results are comparable to those published in these studies and we additionally report a longer follow-up period of 2 years. Moreover, our cohort is representative of an all-comer patient population, which includes various cases of poor prognosis such as active endocarditis. The sole patient who died was in this group and had acquired immunodeficiency syndrome and ongoing sepsis. This technique is versatile and seems to be very appropriate to treat IE even with annular abscess with reconstruction of the ventricular aortic continuity after debridement with additional treated pericardial patches.

**Figure 4:**
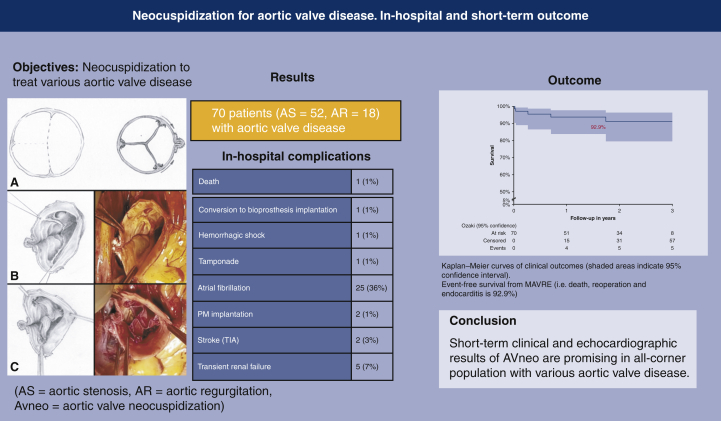
The procedure of aortic valve neocuspidization (*AVneo*), the results obtained in 70 patients with the incidence and the type of in-hospital complications. The outcome (event-free survival) is presented as a survival curve. The event-free survival was 92.1%. *AS*, Aortic stenosis; *AR*, regurgitation; *PM*, pacemaker; *TIA*, transient ischemic attack; *MAVRE*, major adverse valve related event.

There are concerns about in-hospital mortality and incidence of complications due to the complexity of the AVneo procedure. Ozaki and colleagues^15^ described an in-hospital mortality rate of 1.8% in 850 patients. Our study was comparable with a mortality rate of 1.4% at 30 days (Figure 4). Other published studies report rates varying from 0% to 3.3% for mortality at 30 day.^22^^,^^23^ This is very similar to the mortality for surgical AVR.^23^

Our complication rate appears acceptable considering we treated generally more complex patients with almost half of them requiring a combined procedure. This also explains the longer crossclamp and CPB times. Conversely, our patients who only underwent the AVneo procedure showed crossclamp and CPB times comparable to those reported in the literature.^31^, ^22^, ^23^, ^24^ Of particular note is that crossclamp and CPB times were significantly higher if tricuspidization was required, as for BAV.

Additionally, in our study we report 2 permanent pacemaker implantations and 7 new cases of left bundle branch block. Ozaki and colleagues^18^ and Krane and colleagues^23^ reported a lower incidence of conduction abnormalities; however, we performed 9 myomectomies, a well-known risk factor for induction of conduction abnormalities. We also report longer duration of in-hospital stay than usual. This is related to our policy of only letting patients going home when the inflammatory process is normalizing, which takes longer with this type of intervention.

Hemodynamic performance of valve prosthesis is of crucial importance in terms of durability, quality of life, long term outcome, and left ventricle remodeling. In this regard, our mean AVA and mean PPG at discharge were similar to that reported by other groups and remained stable during follow-up, confirming favorable hemodynamic parameters after AVneo, even in patients with a small annulus without any patient–prosthesis mismatch. Moreover, our study showed these parameters to be at least equivalent, if not better, than those typically seen after alternative bioprosthesis implantation.^25^, ^30^, ^26^, ^27^

We did not observe different outcomes between AS and AR groups apart from longer in-hospital stays in the AR group due to the complexity of cases. This indicates that AVneo may be considered for the treatment of a wide range of aortic diseases.

Larger studies on bioprosthesis longevity show that degeneration occurs more quickly in younger patients.^23^, ^28^ However, in all of the studies we reviewed, including ours, patient age was lower than that reported by Ozaki and colleagues.^15^ In our opinion, this points to a possible compelling benefit in proposing the AVneo intervention to the younger patient population.

It is further reported that the rate of freedom from reoperation in patients younger than age 60 years significantly decreases after 15 and 20 years to 70% and 38%, respectively.^24^ It is therefore necessary to conduct studies with longer follow-up to confirm whether or not the AVneo technique should be proposed as a first option in this category of young patients.

Favorable long-term results may be enhanced, in particular, by mitigating various degenerative factors. Excellent hemodynamic parameter results and the origin of implanted material will be important contributors. Autologous tissue offers better biocompatibility than xenogeneic tissues and will improve results in terms of durability. However, the glutaraldehyde used for crosslinking is well known to be cytotoxic, proinflammatory, and is implicated in graft calcification.^26^, ^29^^,^^32^ In at least 1 patient, the histology of neoleaflets removed at 3 months from a patient with AR due to suture release showed fibrinoid and inflammatory reactions with giant inflammatory cells and macrophages with only partial (50%) endothelialization. Consequently, we are currently investigating technical improvements to eliminate glutaraldehyde in favor of less cytotoxic crosslinking agents.

Finally, some have argued that transcatheter AV implantation should not be proposed to patients previously treated with the AVNeo technique due to a need to verify leaflet size and the risk of coronary occlusion. A preliminary case report shows encouraging results of transcatheter AV implantation following AVNeo intervention provided precautions are taken during the transcatheter AV implantation procedure.^33^ Of course, it is too early to draw conclusions and make guidelines for the eventual use of transcatheter AV implantation after AVneo and recurrent AS.

### Study Limitations

First, our study is of relatively short duration. Longer-term studies are needed with follow-up of 10 to 15 years to confirm these encouraging preliminary clinical results. Thus, we have planned to follow our patients long-term. Secondly, our study was a nonrandomized, monocentric study with a relatively small number of patients.

## Conclusions

The AVneo procedure with glutaraldehyde fixed autologous pericardium produced excellent results in terms of survival, operative risk, and valve-related complications at short-term follow-up in adults with a wide range of AVD in our center. Multicenter long-term studies are warranted in the future to confirm the validity of this procedure.

### Conflict of Interest Statement

The authors reported no conflicts of interest.

The *Journal* policy requires editors and reviewers to disclose conflicts of interest and to decline handling or reviewing manuscripts for which they may have a conflict of interest. The editors and reviewers of this article have no conflicts of interest.

## Supplementary Data

Video 1 Video showing the course of the procedure. Video available at: https://www.jtcvs.org/article/S2666-2736(21)00257-6/fulltext.
