# Supplementary figures and images for: Short-term outcomes of aortic valve neocuspidization for various aortic valve diseases
Source: JTCVS Open. 2021 Aug 26;8:193–202. doi: 10.1016/j.xjon.2021.08.027 (PMC9390173; doi:10.1016/j.xjon.2021.08.027)

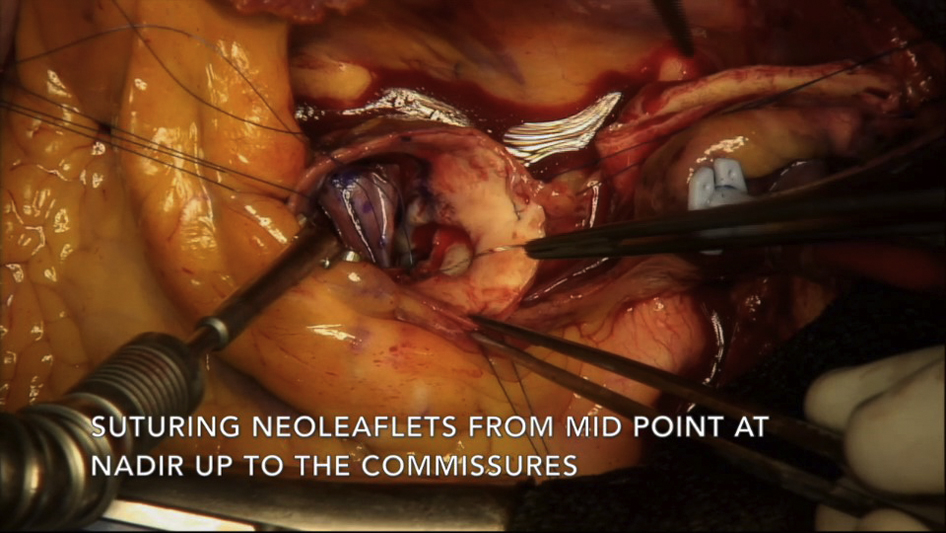

Supplement: Video 1 — Video showing the course of the procedure. Video available at: https://www.jtcvs.org/article/S2666-2736(21)00257-6/fulltext. [file fx3.jpg]
